# Diffuse optical spectroscopic imaging reveals distinct early breast tumor hemodynamic responses to metronomic and maximum tolerated dose regimens

**DOI:** 10.1186/s13058-020-01262-1

**Published:** 2020-03-13

**Authors:** Anup Tank, Hannah M. Peterson, Vivian Pera, Syeda Tabassum, Anais Leproux, Thomas O’Sullivan, Eric Jones, Howard Cabral, Naomi Ko, Rita S. Mehta, Bruce J. Tromberg, Darren Roblyer

**Affiliations:** 1 Department of Biomedical Engineering, Boston University, 44 Cummington Mall, Boston, MA 02215 USA; 2 Department of Electrical Engineering, Boston University, Boston, MA USA; 3 Beckman Laser Institute and Medical Clinic, University of California, Irvine, Irvine, California USA; 4 Department of Electrical Engineering, University of Notre Dame, Notre Dame, IN USA; 5 Department of Biostatistics, Boston University, Boston, MA USA; 6 Department of Hematology and Medical Oncology, Boston Medical Center, Boston, MA USA; 7 Department of Medicine, University of California Irvine, Irvine, California USA

**Keywords:** Diffuse optics, Breast cancer, Treatment monitoring, Metronomic, Neoadjuvant chemotherapy, Flare

## Abstract

**Background:**

Breast cancer patients with early-stage disease are increasingly administered neoadjuvant chemotherapy (NAC) to downstage their tumors prior to surgery. In this setting, approximately 31% of patients fail to respond to therapy. This demonstrates the need for techniques capable of providing personalized feedback about treatment response at the earliest stages of therapy to identify patients likely to benefit from changing treatment. Diffuse optical spectroscopic imaging (DOSI) has emerged as a promising functional imaging technique for NAC monitoring. DOSI uses non-ionizing near-infrared light to provide non-invasive measures of absolute concentrations of tissue chromophores such as oxyhemoglobin. In 2011, we reported a new DOSI prognostic marker, oxyhemoglobin flare: a transient increase in oxyhemoglobin capable of discriminating NAC responders within the first day of treatment. In this follow-up study, DOSI was used to confirm the presence of the flare as well as to investigate whether DOSI markers of NAC response are regimen dependent.

**Methods:**

This dual-center study examined 54 breast tumors receiving NAC measured with DOSI before therapy and the first week following chemotherapy administration. Patients were treated with either a standard of care maximum tolerated dose (MTD) regimen or an investigational metronomic (MET) regimen. Changes in tumor chromophores were tracked throughout the first week and compared to pathologic response and treatment regimen at specific days utilizing generalized estimating equations (GEE).

**Results:**

Within patients receiving MTD therapy, the oxyhemoglobin flare was confirmed as a prognostic DOSI marker for response appearing as soon as day 1 with post hoc GEE analysis demonstrating a difference of 48.77% between responders and non-responders (*p* < 0.0001). Flare was not observed in patients receiving MET therapy. Within all responding patients, the specific treatment was a significant predictor of day 1 changes in oxyhemoglobin, showing a difference of 39.45% (*p* = 0.0010) between patients receiving MTD and MET regimens.

**Conclusions:**

DOSI optical biomarkers are differentially sensitive to MTD and MET regimens at early timepoints suggesting the specific treatment regimen should be considered in future DOSI studies. Additionally, DOSI may help to identify regimen-specific responses in a more personalized manner, potentially providing critical feedback necessary to implement adaptive changes to the treatment strategy.

## Background

Neoadjuvant chemotherapy (NAC) is an important treatment strategy for breast cancer patients with early-stage disease. NAC is used to downstage primary tumors and its use has led to more breast-conserving surgeries [30]. Pathologic complete response (pCR) to NAC, defined as the absence of invasive disease at the time of surgery, has been accepted by the FDA as a surrogate endpoint correlated with clinical benefit [17]. The use of pCR as an endpoint in drug studies has the potential benefit of evaluating efficacy much more rapidly than an endpoint of progression-free survival or overall survival and has recently led to accelerated approval of pertuzumab for use in HER2-positive breast cancer in the NAC setting [15]. Patients that fail to achieve pCR but have a substantial reduction in tumor size (> 50%) also receive therapeutic benefit including a higher rate of breast-conserving surgeries [1]. The combination of these partial responders (PR) with pCR patients represent the cohort most likely to benefit from NAC. Unfortunately, 31% of patients fail to respond to therapy [1]. These are the patients most critical to identify so that their therapy can be altered to avoid ineffective treatment and unwarranted side effects.

The ongoing challenge of highly heterogeneous responses to cancer therapeutics, combined with the increasing array of therapeutic agents and dosing regimens, highlights the importance of tools that can assist oncologists in personalizing, monitoring, and adapting regimens to improve outcomes and limit toxicity. Unfortunately, current methods to assess treatment response, especially at early stages of treatment, are limited [19]. Standard of care imaging modalities such as mammography, ultrasound, and MRI provide anatomical information which has shown limited success in predicting response at early timepoints [26, 34, 52]. Functional imaging modalities such as FDG-PET [2], contrast enhanced MRI [41], and MRS [11] have often demonstrated both earlier and improved prognostic ability, but these modalities suffer from high cost and/or necessity of contrast agents making them impractical for frequent longitudinal monitoring.

Diffuse optical spectroscopic imaging (DOSI) and diffuse optical tomography (DOT) are emerging as affordable, non-invasive functional imaging modalities for longitudinally monitoring breast tumors during NAC [18, 47]. DOSI uses near-infrared light (650–1000 nm) to interrogate tissue optical absorption and scattering properties up to several centimeters in depth [4, 40, 43]. DOSI measures absolute concentrations of oxyhemoglobin, deoxyhemoglobin, lipid, and water. These parameters have been shown by multiple research groups to be valuable prognostic markers at various points throughout NAC [6, 9, 16, 21, 27, 45, 46, 48, 49, 50, 53]. For example, a recent landmark multicenter study (ACRIN 6691) showed that changes in tumor deoxyhemoglobin, water, and lipid at midpoint of NAC correlated strongly to pCR [48]. Additionally, we have previously shown that alterations in tumor oxyhemoglobin during the first day of therapy can discriminate responding from non-responding NAC patients, representing the earliest DOSI marker of treatment response to date [45].

Importantly, almost all prior treatment monitoring studies with DOSI and DOT have analyzed NAC treatment response irrespective of regimen, representing a substantial limitation in the field. The magnitude of antiangiogenesis, hypoxia, immune activation, and other biological effects induced by systemic therapies are highly dependent on the specific agents and treatment schedules [3, 28, 50]. Consequently, DOSI and DOT prognostic biomarkers may be highly dependent on the particular agents and schedule. Current standard of care NAC regimens most commonly utilize maximum tolerated dosing (MTD) [37]. MTD is defined as the highest dose that can be administered without unacceptable side effects. This strategy relies on the kinetics of large doses of drug administrations leading to dramatic tumor cytotoxicity, typically followed by a rest period in which the host recovers from off-target effects [22]. In this paradigm, non-responding patients can endure up to several months of severe cytotoxic side effects without any therapeutic benefit. Alternative treatment dosing schedules are currently being explored to both enhance therapeutic efficacy and reduce off-target toxicity. Perhaps most importantly, metronomic (MET) scheduling, which utilizes lower-dose agents administered more frequently, has demonstrated promising anti-angiogenic properties [29, 42] as well as antitumor immune activation while limiting side effects [28, 33, 36]. There continues to be considerable interest in investigating whether MET regimens can improve response rates and outcomes in breast and other cancers, demonstrated by the dozens of active clinical studies exploring metronomic regimens listed on www.clinicaltrials.gov.

We present here clinical evidence of regimen-specific DOSI response during early NAC, demonstrating that the oxyhemoglobin flare, a transient increase in tumor oxyhemoglobin concentration occurring during the first week of NAC, manifests as a powerful prognostic marker in patients receiving an MTD regimen, but fails to appear in a well-matched MET cohort. In MTD patients, oxyhemoglobin flare peaked 24 h following the first chemotherapy infusion. This work highlights the importance of precision treatment monitoring strategies that account for the specific therapeutic regimen.

## Methods

### Spectroscopy

Specific details about the DOSI instrumentation have been well described elsewhere [4]. Briefly, DOSI uses near-infrared light (650–1000 nm) to measure deep tissue functional information with a handheld probe. For this study, fiber-coupled temporally modulated laser diodes (659, 689, 781, 829 nm or 658, 682, 785, 810, 830, 850 nm) and a broadband near-infrared light source (650–1000 nm) were used to illuminate the tissue from the skin surface. An avalanche photodiode was used to detect the remitted temporally modulated laser light and a spectrometer was used to measure the remitted broadband light. The lasers were frequency swept from 50 to 400 MHz; the amplitude and phase perturbations induced by the tissue were measured with a vector network analyzer or custom analog electronics [39]. The amplitude and phase at each wavelength and modulation frequency were fit to an analytical solution to the P1 diffusion approximation of the Boltzmann transport equation solved in the frequency-domain with semi-infinite boundary conditions [24]. This information was combined with broadband diffuse reflectance measurements to yield broadband optical absorption and reduced scattering properties [4]. Absolute concentrations of oxyhemoglobin (HbO_2_), deoxyhemoglobin (HHb), water, and lipid were then determined by a least squares fitting procedure of the broadband absorption spectra to the known extinction coefficient spectra of these four chromophores according to Beer’s law. Additional composite metrics were also computed including total hemoglobin (THb = HbO_2_ + HHb), oxygen saturation (StO_2_ = HbO_2_ /THb), and tissue optical index (TOI = HHb × water/lipid). All data analysis was conducted using custom processing codes developed in MATLAB R2014b (MathWorks Inc).

### Imaging procedure

**Fig. 1:**
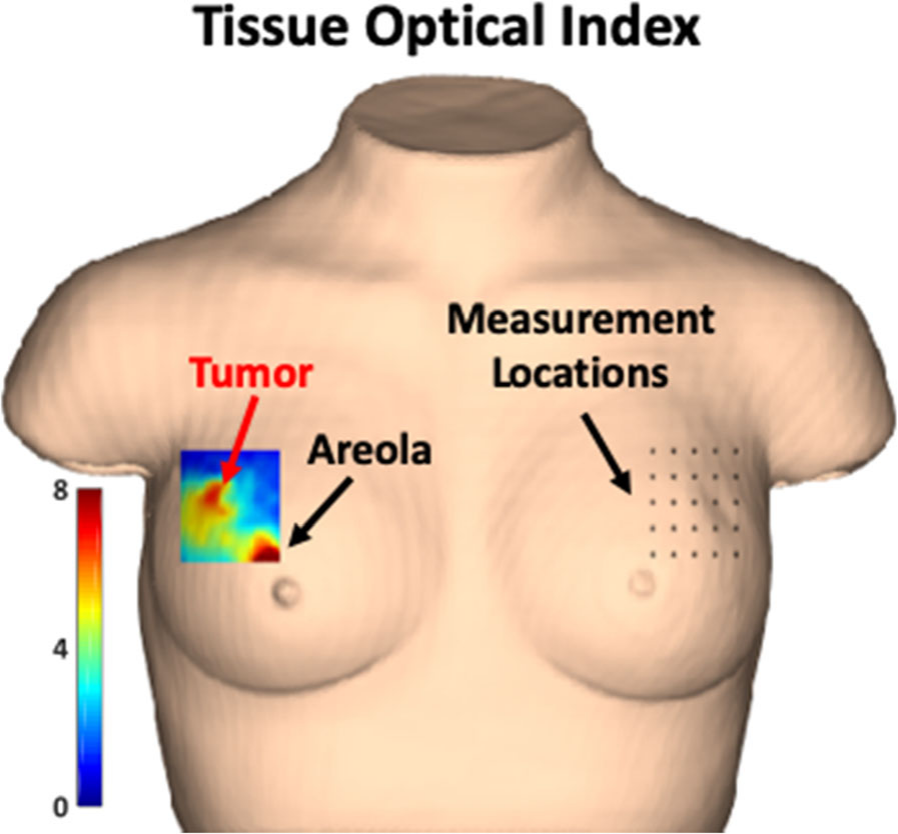
Bust model of DOSI measurement. Example of DOSI measurement grid locations (right) and subsequent TOI map (left) demonstrating tumor and areolar contrast

DOSI scans were conducted prior to chemotherapy administration (baseline) and as many days as possible within the first week of starting therapy dependent on patient availability and health status. A standardized DOSI measurement protocol was used to measure the patients [48]. Briefly, a sequential rectangular grid pattern with 1-cm spacing was transferred to the tissue using a transparency and surgical marker. The DOSI probe was placed against the breast and a measurement was taken at every point on the grid. Landmarks such as the nipple, areola, freckles, and moles were used to coregister longitudinal measurements. The size of the rectangular grid was chosen to fully include the tumor region with clear margins as determined by prior standard of care imaging and palpation. An example of a measurement grid is shown in Fig. 1 on the left breast using a 3D bust model. An interpolated heatmap of the TOI composite metric in shown over the right breast over a 3.7-cm tumor. The tumor and areola both show significant TOI contrast relative to the surrounding breast tissue, a highly conserved feature seen across many breast cancer subjects [7].

### Image analysis

2D heatmaps of each chromophore (i.e., HbO_2_, HHb, water, lipid) and composite metrics (i.e., THb, StO_2_, TOI) were generated for each subject and at each time point. A tumor region of interest (ROI) was determined using a combination of peak TOI contrast, as described by previous studies [6, 7, 48], and tumor size was determined by ultrasound or MRI. The tumor ROI size remained constant throughout the longitudinal measurements but was allowed to laterally shift in cases of grid displacement. Tumor chromophore concentrations were calculated by taking the mean over the tumor ROI.

### Subject eligibility and enrollment

A total of 53 female breast cancer patients were measured at Boston Medical Center (BMC) and University of California, Irvine (UCI) from May 2004 to October 2017. Subject’s ages ranged from 26 to 71. One patient had bilateral breast cancer with both tumors measured for a total of 54 DOSI monitored tumors. Eligible subjects had a diagnosis of invasive breast cancer and were planned for neoadjuvant cytotoxic chemotherapy with definitive breast surgery following therapy. Each patient’s treatment regimen was determined by their oncologist; a subset of subjects was simultaneously enrolled in an investigational protocol testing MET dosing and scheduling (Clinicaltrials.gov Identifier NCT00618657). The patients received a biopsy prior to treatment to confirm invasive cancer diagnosis, which provided the tumor receptor status (i.e., ER, PR, HER2). Potential subjects were excluded if they were pregnant or previously received treatment to the affected breast. Data from a portion of these 54 subjects had been used in prior published works, including the initial observation of oxyhemoglobin flare [45], which analyzed 24 of the current 54 subjects throughout week 1, including day 1. The remaining 30 subjects’ data at day 1 have not been published before, although 14 of the current 54 subjects were also enrolled in the ACRIN 6691 study [10, 48], which included baseline, early (days 5–10), midpoint, and pre-surgical timepoint measurements. All patients provided written informed consent. This project was approved by the Institution Review Board at Boston University, Boston Medical Center and UCI.

### Histopathology

Each patient’s resected tumor was evaluated by the local institution’s pathologist to generate a pathology report. After treatment, the patient was assigned a tertiary response status according to the National Surgical Adjuvant Breast and Bowel Project Protocol [44]: pathologic complete response (pCR), partial response (PR), and no response (NR). pCR was defined as no residual tumor burden in resected tumor. PR was defined as a decrease in the largest tumor dimension by > 50% from diagnosis to resection while NR was defined as a decrease of < 50%. A binary classification of response was also utilized in which responders, defined as subjects achieving either pCR or PR, were compared against NR subjects.

### Statistical analysis

Generalized estimating equations (GEE) were used to longitudinally model the DOSI chromophores throughout the first week after chemotherapy utilizing SAS (SAS Institute) [31]. The GEE accounted for the correlation between multiple measurements on a single patient and allowed for an unbalanced dataset with subjects considered as clusters, an exchangeable correlation structure, and a normal model with an identity link function. Separate models were run on each of four chromophores: HbO_2_, HHb, water, and lipid. In addition, for each outcome variable, three separate models were run on population stratifications of treatment schedule and pathologic response to isolate the effects of specific covariates and interaction terms: (1) MTD responders vs MTD non-responders, (2) MET responders vs MET non-responders, and (3) MTD responders vs MET responders. Additional covariates included in these models included as follows: institution (Boston Medical Center vs UC Irvine), age, hormone receptor status (estrogen receptor or progesterone receptor), and HER2 status. Significance for model parameters was determined at a level of 0.05 and when adjusted for multiple comparisons with Bonferroni correction at a level of 0.0125. Additional covariates relating to treatment such as chemotherapeutic agent or drug mechanism were not included as these parameters were highly correlated with treatment schedule (e.g., all patients administered adriamycin + cyclophosphamide received an MTD schedule). Post hoc contrasts between outcome means adjusted for covariates in the statistical models were performed at each day post chemotherapy between strata of interest: (1) MTD: responders vs non-responders and (2) responders: MTD vs MET. Significance for post hoc contrasts, when adjusted for multiple comparisons with Bonferroni correction, was determined at a level of 0.0036.

Linear discriminant analysis (LDA) was performed using MATLAB to assess the prognostic accuracy of percent change in oxyhemoglobin on day 1 among MTD and MET population. This analysis assumed multivariate normal densities and equal covariance for each group. Fivefold cross-validation was used to train and test the dataset and limit overfitting. We note that there was approximately twice as many responders as non-responders in the MTD cohort and no efforts were made to account for this imbalance in the classification analysis. Posterior probabilities were calculated for each subject from the linear classification of responders from non-responders. Receiver operating characteristic (ROC) curves were generated by iterating through all posterior probability thresholds. The area under the curve (AUC) of the ROC curve was utilized to evaluate the performance of the model along with the optimal sensitivity, specificity, positive predictive value (PPV), and negative predictive value (NPV).

## Results

### Subject and treatment characteristics

The characteristics of all analyzed subjects are shown in Table 1, which shows the overall population (*n* = 54) statistics as well as characteristics stratified by treatment schedule: maximum tolerated dose (MTD, *n* = 35) and metronomic (MET, *n* = 19). The MTD patients received treatment every 2 or 3 weeks at the highest dose without unacceptable side effects per the standard of care. In contrast, MET patients received treatment every week at a smaller dose than MTD. The average overall subject age was 49.9 ± 11.1 years with a slightly higher age among the MTD subset, 51.3 ± 11.1 years and slightly lower in MET subset, 47.4 ± 10.8 years. The average tumor size was 3.5 ± 1.7 cm for all subjects, 3.8 ± 1.9 cm for MTD subjects, and with 3.0 ± 1.3 cm MET subjects. Age and tumor size were not significantly different (*p* > 0.05) between the MTD and MET cohort through the Wilcoxon rank sum test using MATLAB, utilizing a nonparametric test to avoid any assumptions of the underlying distributions.

**Table 1:** Subject and tumor characteristics

Variables	Treatment cohorts		
	Maximum tolerable dose (*n* = 35)	Metronomic dose (*n* = 19)	Overall (*n* = 54)
Age (years)			
Mean ± SD	51.3 ± 11.1	47.4 ± 10.8	49.9 ± 11.1
Tumor Size (cm)			
Mean ± SD	3.8 ± 1.9	3.0 ± 1.3	3.5 ± 1.7
Location			
Left	15 (43%)	10 (53%)	25 (46%)
Right	20 (57%)	9 (47%)	29 (54%)
Histology			
IDC	31 (89%)	18 (95%)	49 (91%)
ILC	4 (11%)	1 (5%)	5 (9%)
Receptor status			
ER			
−	13 (37%)	5 (26%)	18 (33%)
+	21 (60%)	14 (74%)	35 (65%)
PR			
−	15 (42%)	8 (42%)	23 (43%)
+	19 (54%)	11 (58%)	30 (56%)
HER2			
−	24 (69%)	14 (74%)	38 (70%)
+	10 (29%)	5 (26%)	15 (28%)
Unknown receptor (ER,PR,HER2)	1 (2%)	0	1 (2%)
Pathologic response			
Complete response	10 (29%)	5 (26%)	15 (28%)
Partial response	15 (43%)	5 (26%)	20 (37%)
No response	10 (29%)	9 (47%)	19 (35%)
Treatment			
AC	30 (86%)	0	30 (56%)
AC + DTX	3 (9%)	0	3 (6%)
Cb + DTX + Tr	1 (3%)	0	1 (2%)
Cb + nPTX+Bev	0	14 (74%)	14 (26%)
Cb + nPTX+Tr	0	5 (26%)	5 (9%)
Pzb + DTX + Tr	1 (3%)	0	1 (2%)
Institute			
UC Irvine	29 (83%)	19 (100%)	48 (89%)
Boston Medical Center	6 (17%)	0	6 (11%)

*Abbreviations*: *IDC* invasive ductal carcinoma, *ILC* invasive lobular carcinoma, *ER* estrogen receptor, *PR* progesterone receptor, *HER2* Human Epidermal Growth Factor Receptor 2, *AC* adriamycin and cyclophosphamide, *DTX* docetaxel, *Cb* carboplatin, *Tr* trastuzumab, *nPTX* paclitaxel, *Bev* bevacizumab, *Pzb* pertuzumab

All patients received a diagnosis of invasive carcinoma with the majority of patients (91%) receiving a diagnosis of invasive ductal carcinoma (IDC). In total, 65% of subjects had hormone receptor-positive tumors. Fifteen subjects were diagnosed with HER2-positive tumors; of those, seven received trastuzumab (Tr) as part of their first neoadjuvant treatment and the other eight received Tr at a timepoint which was not in the scope of this study. Within the MTD treatment schedule, one patient received a combination of HER2-targeted therapies with both Tr and pertuzumab (Pzb) along with the cytotoxic agent docetaxel (DTX). One MTD patient received a combination of carboplatin (Cb), DTX, and Tr. Three MTD patients received adriamycin (A) + cyclophosphamide (C) + DTX, while 30 MTD patients received A + C, which is the current standard of care therapy. Among the MET patients, 14 patients received carboplatin (Cb) + paclitaxel (nPTX) + bevacizumab (Bev) and 5 patients received Cb + nPTX + Tr.

At the time of surgery, 35 patients (65%) were determined to be responders while 19 patients (35%) were non-responders. A total of 71% of MTD patients were responders (*n* = 25) and 53% of MET patients were responders (*n* = 10). All proportions (location, histology, receptor status, and pathologic response) were not significantly different between MTD and MET cohorts (*p* > 0.05) using the *Z*-Test for proportions implemented in MATLAB.

### DOSI reveals response and regimen-dependent HbO_2_ changes on day 1 of NAC

**Fig. 2:**
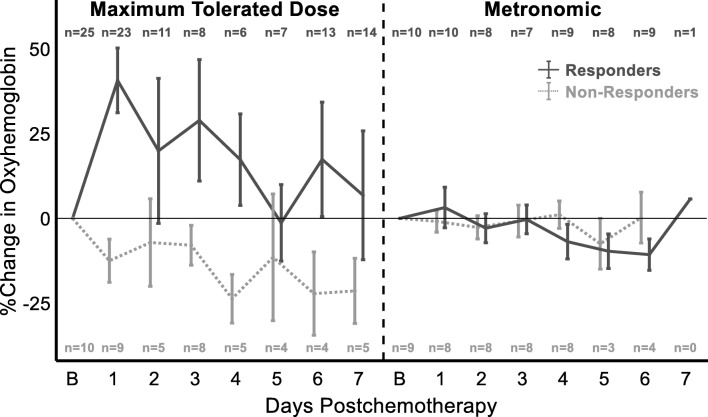
Week 1 postchemotherapy changes of DOSI-monitored tumors. Percent change in oxyhemoglobin during the first week postchemotherapy separated by treatment: maximum tolerated dose (left) and metronomic (right) and pathologic response: responders (dark gray, solid line) and non-responders (light gray, dashed). At day 1, MTD responders and non-responders are significantly different (*p* < 0.0001), while MET subjects fail to demonstrate a statistical difference. Number of subjects measured at each timepoint is indicated and color-coded for pathologic response. Error bars indicate mean ± standard error

**Fig. 3:**
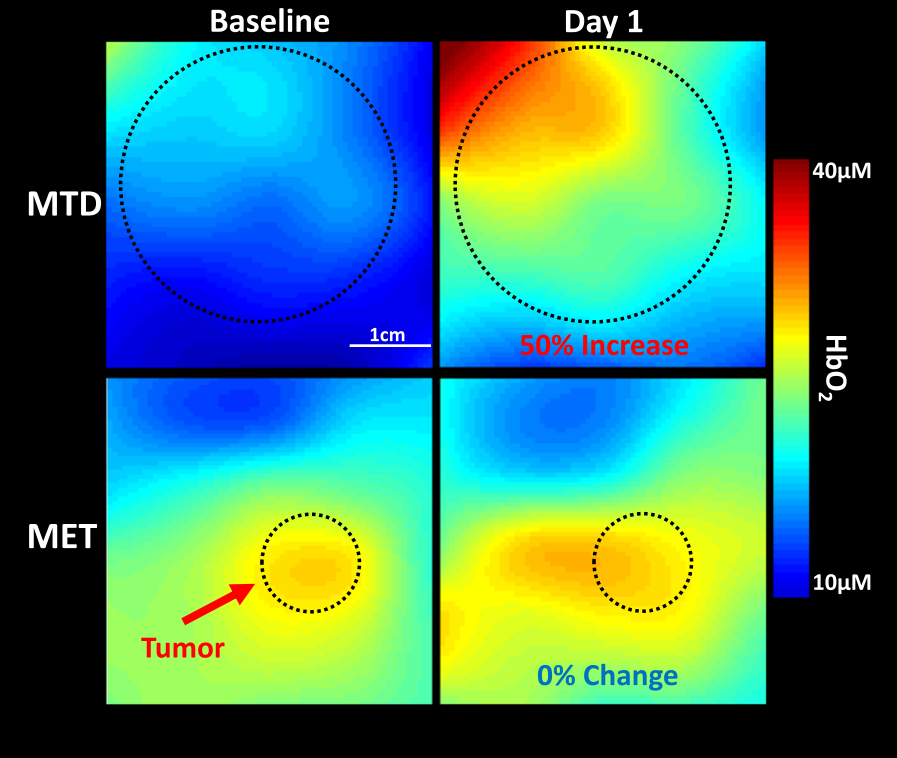
Map of oxyhemoglobin flare and regimen-specific response. Interpolated HbO_2_ maps at baseline (left column) and day 1 (right column) for two pathologic complete responder patients receiving maximum tolerated dose (top) and metronomic (bottom). The tumor region is indicated by the dashed circle with the scale bar indicating 1 cm. The percent change from baseline for the entire tumor region is indicated in the day 1 column showing oxyhemoglobin flare in MTD patient while MET patient showed almost no change

The percent change from baseline was examined to normalize for varying baseline tumor chromophore concentrations among patients, with a focus on the primary aim of day 1 postchemotherapy changes. The observed changes across week 1 are shown in Fig. 2. For subjects receiving MTD treatment, the largest difference between responders and non-responders occurred on day 1. Responders on day 1 demonstrated a mean 40% increase in HbO_2_ at day 1 compared to a 13% decrease in non-responders. All MET subjects displayed much smaller changes on day 1: responders with a 3% increase and non-responders with a 1% decrease. These differences are visualized in Fig. 3, which shows representative 2D DOSI heatmaps of HbO_2_ concentrations at both baseline and day 1 for two different pCR subjects, one of whom received MTD treatment while the other received MET therapy. The MTD patient had a large increase of 50% in HbO_2_ from baseline to day 1 contrasted with the MET patient, which only increased by 0.3%. In addition, there were relatively small changes in both MET responders and non-responders across the entire week 1 as compared to the MTD patients.

Separate GEE models were fit for the MTD and MET subject populations to isolate the effect of treatment and evaluate the effects of age, institution, hormone receptor status, HER2 status, days post chemotherapy, response, and the corresponding interaction terms. Within the MTD cohort, the interaction term of response and day 1 was a significant predictor of HbO_2_ (*p* < 0.0001) while the MET cohort failed to demonstrate a statistically significant predictor. In order to isolate the effect of pathologic response, a separate GEE model was run on the responder population to determine the effects of age, institution, hormone receptor status, HER2 status, days post chemotherapy, treatment schedule, and the corresponding interaction terms. The interaction term of treatment schedule and day 1 was a significant predictor of HbO_2_ (*p* = 0.0008) in responders. Post hoc analysis of outcome adjusted estimates to compare the effect of treatment demonstrated a significant difference in day 1 HbO_2_ changes between MTD responders and MET responders (39.45 ± 11.98%, *p* = 0.0010) seen in Additional file 1: Table S1A. Similar analysis on the efficacy of HbO_2_ as a prognostic biomarker at day 1 for patients receiving MTD demonstrated a difference of 48.77 ± 9.51% (*p* < 0.0001) between responders and non-responders seen in Additional file 1: Table S1B. None of the additional covariates (age, institution, hormone receptor status, HER2 receptor status) contributed significantly to the model.

Separate LDAs were run on the MTD and MET subject populations to evaluate HbO_2_ performance on day 1 as a prognostic predictor when isolating the effect of treatment, results shown in Additional file 2: Figure S1. The MTD cohort had an AUC of 0.89, sensitivity of 0.91, specificity of 0.89, PPV of 0.95, and NPV of 0.80. In stark contrast, the MET cohort had an AUC of 0.50, sensitivity of 0.50, specificity of 0.75, PPV of 0.71, and NPV of 0.55.

### DOSI measured changes in HHb, water, and lipid throughout week 1 of NAC

HHb, water, and lipid did not exhibit significant prognostic or regimen-dependent changes in either MTD or MET patients. GEE analysis was run on these chromophores for each of the three population stratifications and there were no significant interactions of treatment or response at any day. The most notable interaction term was response and day 1 post chemotherapy (*p* = 0.0136) for water in MTD patients, which came close to significance (*p* < 0.0125 for Bonferroni correction). At this timepoint, non-responding MTD patients had a moderate decrease in water concentration compared to responders (Additional file 3: Figure S2).

## Discussion

Early prediction of NAC response would provide important feedback to alter the therapeutic regimen for each individual patient. We have demonstrated here that early DOS imaging markers are dependent on the specific NAC regimen administered. Specifically, MTD and MET regimens resulted in significantly different oxyhemoglobin responses throughout week 1 of NAC. Oxyhemoglobin flare, a previously reported prognostic biomarker for therapy response, manifested only in MTD-treated breast tumors, while MET treatment yielded almost no hemodynamic changes across throughout week 1, regardless of response.

There have been few clinical monitoring studies of breast cancer patients at the earliest timepoints of neoadjuvant chemotherapy. Clinical studies utilizing magnetic resonance spectroscopy [35] and FDG-PET [12] have previously observed a prognostic metabolic change within 1 day of therapy. The authors of the MRS study hypothesized that responding tumors receiving AC treatment underwent cytotoxicity and/or decreases in tumor proliferation at day 1, while the authors of the FDG-PET study demonstrated increased metabolic activity in responsive tumors on day 1. Our group has previously demonstrated the presence of the oxyhemoglobin flare on day 1, which provided discrimination of responders from non-responders in a cohort of 24 subjects, 21 of whom received MTD regimens [45]. The present study included data from these prior subjects with the addition of 14 additional MTD subjects and 16 additional MET subjects. Eleven new/previously unpublished MTD patients were measured on day 1, of which 3/5 responders displayed flare and 5/6 non-responders did not display flare, (no increase in oxyhemoglobin). In aggregate of all MTD patients, HbO_2_ served as a strong predictor of NAC response with an AUC of 0.89.

Most strikingly, the oxyhemoglobin flare was only present in the subjects who received an MTD regimen, failing to manifest in subjects treated with a MET regimen and serving as a poor predictor of response with an AUC of 0.50. In MET subjects, only small (< 11%) changes in oxyhemoglobin were observed across the entire week 1 of therapy and no prognostic changes occurred in any of the other hemodynamic parameters including deoxyhemoglobin, total hemoglobin, and oxygen saturation. The presence of oxyhemoglobin flare in responding MTD subjects and a lack of hemodynamic response in both non-responding MTD subjects and all MET subjects suggest a distinct physiological reaction in responding MTD patients. A rise in oxyhemoglobin may occur due to either a decrease in tumor oxygen demand or an increase in tumor oxygen supply. Subjects that experienced flare had a relatively small change in deoxyhemoglobin leading to an increase in total hemoglobin and oxygen saturation (Additional file 3: Figure S2), indicating an increase in oxygen supply. This would be consistent with the increased perfusion known to co-occur with an inflammatory response to cytotoxic cellular damage [5, 20, 25, 32, 38, 51]. The lack of oxyhemoglobin flare in MTD NR patients may indicate poor chemo- and/or immuno-responsiveness, leading to minimal shrinkage of the tumor and potentially poorer outcomes [13, 23].

MET therapy is characterized by the administration of lower-dosage therapy with increased frequency. In this study, the low initial dose may have been insufficient to induce oxyhemoglobin flare, even in subjects who went on to achieve partial or complete responses. Additionally, MET regimens have been shown to exhibit substantially different anti-angiogenic and immunomodulatory effects compared to MTD [3, 8, 14, 22, 28, 36, 42]. Additionally, 14 of the 19 MET subjects received the VEGF-A targeting drug bevacizumab in addition to cytotoxic therapies. Clinical administration of bevacizumab as a monotherapy has been shown to cause hypoxia and decreased total hemoglobin on day 1 when measured with a time-domain diffuse optical system [50]. It is possible the administration of bevacizumab inhibited oxyhemoglobin flare in responding MET subjects. These varying mechanisms of actions may help to explain the lack of early hemodynamic responses observed in MET patients in this study. Notably, several other publications [6, 10, 48, 49] included subjects enrolled in the same investigational MET trial, although the DOSI measurement timepoints investigated in these studies did not include day 1 measurements.

This retrospective study did not control for chemotherapeutic agents as patients across and in the MTD and MET cohorts received different agents as well as scheduling and dosing. The standard of care for NAC allows for flexibility in the order of agents and the exact regimen, determined by the oncologist. This further demonstrates the necessity to account for all aspects of the treatment regimen when investigating prognostic biomarkers. The combination of different agents and dosing strategies may yield different synergistic mechanisms of action, ultimately affecting the induction of oxyhemoglobin flare. Within the MTD cohort, most patients received AC; however, some received additional DTX, representing a potential confounding variable. In the MET cohort, patients were given differing targeted agents, either Bev or Tr. Secondary methods may be necessary to confirm the exact biological origins of the flare through inflammatory and immune markers. Additional potential future analysis steps include a *Z*-score analysis like that conducted by Cochran et al. in their analysis of DOSI measurements at early timepoints (within days 5–10 of the start of treatment), as well as determining if treatment schedule plays a significant factor at later DOSI measurement timepoints [10].

## Conclusion

In summary, we have demonstrated regimen-dependent hemodynamic responses during week 1 of NAC. Oxyhemoglobin flare manifested as a prognostic marker only in an MTD cohort and not in a MET cohort. This is important as DOSI and DOT treatment monitoring studies have traditionally aggregated subjects irrespective of treatment, while different regimens have markedly different biological mechanisms of action. Early regimen-specific DOSI prognostic markers could be critical for improving patient outcome by identifying non-responders and adapting therapy accordingly. DOSI may also provide valuable feedback of investigational drug regimens and their proposed mechanisms of action.

## Supplementary information

Additional file 1. Post-hoc GEE Analysis. A) Post-hoc GEE Estimates of Oxyhemoglobin differences across Response of MTD patients on days 1-7. B) Post-hoc GEE Estimates of Oxyhemoglobin differences across Treatment of Responding patients on days 1-7. Significance is determined at Bonferroni corrected level of *p*<0.0036.

Additional file 2. Prognostic Accuracy of Oxyhemoglobin Flare. Receiver Operator Characteristic (ROC) Curve of percent change in oxyhemoglobin on day 1 postchemotherapy as a classifier for pathologic response (Responders vs Non-Responders) for both MTD (dark grey) and MET (light grey) with their corresponding area under the curve (AUC).

Additional file 3. Day 1 Hemodynamic Changes of DOSI-monitored tumors. Percent change during day 1 postchemotherapy in oxyhemoglobin, deoxyhemoglobin, oxygen saturation, total hemoglobin, water, and lipid separated by treatment: Maximum Tolerated Dose (left) and Metronomic (right) and pathologic response: Responders (Dark Grey) and Non-Responders (Light Grey). Error bars represent mean ± standard error.

## Data Availability

This data that supports the findings of this manuscript can be made available from the corresponding author following reasonable request.

## Abbreviations

AC: Adriamycin + cyclophosphamide
ACRIN: American College of Radiology Imaging Network
Bev: Bevacizumab
Cb: Carboplatin
DOSI: Diffuse optical spectroscopic imaging
DOT: Diffuse optical tomography
DTX: Docetaxel
GEE: Generalized estimating equations
LDA: Linear discriminant analysis
MET: Metronomic
MTD: Maximum tolerated dose
nPTX: Paclitaxel
Pzb: Pertuzumab
TOI: Tissue optical index
Tr: Trastuzumab

## References

[1] Asselain B, Barlow W, Bartlett J, Bergh J, Bergsten-Nordström E, Bliss J, Boccardo F (2018). Long-term outcomes for neoadjuvant versus adjuvant chemotherapy in early breast cancer: meta-analysis of individual patient data from ten randomised trials. Lancet Oncol.

[2] Avril S, Muzic RF, Plecha D, Traughber BJ, Vinayak S, Avril N (2016). 18F-FDG PET/CT for monitoring of treatment response in breast cancer. J Nucl Med.

[3] Bertolini F, Paul S, Mancuso P, Monestiroli S, Gobbi A, Shaked Y, Kerbel RS (2003). Maximum tolerable dose and low-dose metronomic chemotherapy have opposite effects on the mobilization and viability of circulating endothelial progenitor cells. Cancer Res.

[4] Bevilacqua F, Berger AJ, Cerussi AE, Jakubowski D, Tromberg BJ (2000). Broadband absorption spectroscopy in turbid media by combined frequency-domain and steady-state methods. Appl Opt.

[5] Bracci L, Schiavoni G, Sistigu A, Belardelli F (2014). Immune-based mechanisms of cytotoxic chemotherapy: implications for the design of novel and rationale-based combined treatments against cancer. Cell Death Differ.

[6] Cerussi AE, Tanamai VW, Hsiang D, Butler J, Mehta RS, Tromberg BJ (2011). Diffuse optical spectroscopic imaging correlates with final pathological response in breast cancer neoadjuvant chemotherapy. Philos Trans R Soc A Math Phys Eng Sci.

[7] Cerussi A, Shah N, Hsiang D, Durkin A, Butler J, Tromberg BJ (2006). In vivo absorption, scattering, and physiologic properties of 58 malignant breast tumors determined by broadband diffuse optical spectroscopy. J Biomed Opt.

[8] Chen C-S, Doloff JC, Waxman DJ (2015). Intermittent metronomic drug schedule is essential for activating antitumor innate immunity and tumor Xenograft regression. Neoplasia.

[9] Cochran JM, Chung SH, Leproux A, Baker WB, Busch DR, DeMichele AM, Tchou J, Tromberg BJ, Yodh AG (2017). Longitudinal optical monitoring of blood flow in breast tumors during neoadjuvant chemotherapy. Phys Med Biol.

[10] Cochran JM, Busch DR, Leproux A, Zheng Z, O’Sullivan TD, Cerussi AE, Carpenter PM (2018). Tissue oxygen saturation predicts response to breast cancer neoadjuvant chemotherapy within 10 days of treatment. J Biomed Opt.

[11] Danishada KKA, Sharmaa U, Saha RG, Seenub V, Parshadb R, Jagannathan NR (2010). Assessment of therapeutic response of locally advanced breast cancer (LABC) patients undergoing neoadjuvant chemotherapy (NACT) monitored using sequential magnetic resonance spectroscopic imaging (MRSI). NMR Biomed.

[12] Dehdashti F, Mortimer JE, Trinkaus K, Naughton MJ, Ellis M, Katzenellenbogen JA, Welch MJ, Siegel BA (2009). PET-based estradiol challenge as a predictive biomarker of response to endocrine therapy in women with estrogen-receptor-positive breast cancer. Breast Cancer Res Treat.

[13] Dieci MV, Radosevic-Robin N, Fineberg S, van den Eynden G, Ternes N, Penault-Llorca F, Pruneri G (2018). Update on tumor-infiltrating lymphocytes (TILs) in breast cancer, including recommendations to assess tils in residual disease after neoadjuvant therapy and in carcinoma in situ: a report of the International Immuno-Oncology Biomarker Working Group on Bre. Semin Cancer Biol.

[14] Doloff JC, Waxman DJ (2012). VEGF receptor inhibitors block the ability of metronomically dosed cyclophosphamide to activate innate immunity-induced tumor regression. Cancer Res.

[15] Esserman LJ, DeMichele A (2014). Accelerated approval for pertuzumab in the neoadjuvant setting: winds of change?. Clin Cancer Res.

[16] Falou O, Soliman H, Sadeghi-Naini A, Iradji S, Lemon-Wong S, Zubovits J, Spayne J (2014). Diffuse optical spectroscopy evaluation of treatment response in women with locally advanced breast cancer receiving neoadjuvant chemotherapy. Transl Oncol.

[17] FDA. 2014. “Guidance for industry: pathologic complete response in early-stage breast cancer: use as an endpoint to support accelerated approval.” https://www.fda.gov/media/83507/download. Accessed 27 June 2019.

[18] Gibson A, Dehghani H (2009). Diffuse optical imaging. Philos Trans R Soc A Math Phys Eng Sci.

[19] Graham LJ, Shupe MP, Schneble EJ, Flynt FL, Clemenshaw MN, Kirkpatrick AD, Gallagher C (2014). Current approaches and challenges in monitoring treatment responses in breast cancer. J Cancer.

[20] Grivennikov SI, Greten FR, Karin M (2010). Immunity, inflammation, and Cancer. Cell.

[21] Gunther, Jacqueline E., Emerson A. Lim, Hyun K. Kim, Molly Flexman, Mirella Altoé, Jessica A. Campbell, Hanina Hibshoosh, et al. 2018. “Dynamic diffuse optical tomography for monitoring neoadjuvant chemotherapy in patients with breast cancer.” Radiology 000 (0): 161041. doi: 10.1148/radiol.2018161041. 10.1148/radiol.2018161041 PMC5978455 29431574

[22] Hanahan D, Bergers G, Bergsland E. Less is, more, regularly: metronomic dosing of cytotoxic drugs can target tumor angiogenesis in mice. J Clin Investig. 2000; 10.1172/JCI9872. 10.1172/JCI9872 PMC300842 10772648

[23] Hanahan D, Weinberg RA (2011). Hallmarks of cancer: the next generation. Cell.

[24] Haskell RC, Svaasand LO, Tsay T-T, Feng T-C, Tromberg BJ, McAdams MS (1994). Boundary conditions for the diffusion equation in radiative transfer. J Opt Soc Am A.

[25] Hendry SA, Farnsworth RH, Solomon B, Achen MG, Stacker SA, Fox SB (2016). The role of the tumor vasculature in the host immune response: implications for therapeutic strategies targeting the tumor microenvironment. Front Immunol.

[26] Hylton NM, Blume JD, Bernreuter WK, Pisano ED, Rosen MA, Morris EA, Weatherall PT (2012). Locally advanced breast cancer: MR imaging for prediction of response to neoadjuvant chemotherapy—results from ACRIN 6657/I-SPY TRIAL. Radiology.

[27] Jiang S, Pogue BW, Kaufman PA, Gui J, Jermyn M, Frazee TE, Poplack SP, DiFlorio-Alexander R, Wells WA, Paulsen KD (2014). Predicting breast tumor response to neoadjuvant chemotherapy with diffuse optical spectroscopic tomography prior to treatment. Clin Cancer Res.

[28] Kareva I, Waxman DJ, Lakka G (2015). Metronomic chemotherapy : an attractive alternative to maximum tolerated dose therapy that can activate anti-tumor immunity and minimize therapeutic resistance. Cancer Lett.

[29] Kerbel RS, Kamen BA (2004). The anti-angiogenic basis of metronomic chemotherapy. Nat Rev Cancer.

[30] Kümmel S, Holtschmidt J, Loibl S (2014). Surgical treatment of primary breast cancer in the neoadjuvant setting. Br J Surg.

[31] Littell R, Freund R, Spector P (1991). SAS System for Linear Models.

[32] Martin JD, Seano G, Jain RK (2019). Normalizing function of tumor vessels: Progress, opportunities, and challenges. Annu Rev Physiol.

[33] Masuda N, Higaki K, Takano T, Matsunami N, Morimoto T, Ohtani S, Mizutani M (2014). A phase II study of metronomic paclitaxel/cyclophosphamide/capecitabine followed by 5-fluorouracil/epirubicin/cyclophosphamide as preoperative chemotherapy for triple-negative or low hormone receptor expressing/HER2-negative primary breast cancer. Cancer Chemother Pharmacol.

[34] Mclaughlin R, Hylton N (2011). MRI in breast cancer therapy monitoring. NMR Biomed.

[35] Meisamy S, Bolan PJ, Baker EH, Bliss RL, Gulbahce E, Everson LI, Nelson MT (2004). Neoadjuvant chemotherapy of locally advanced breast cancer: predicting response with in vivo 1 H MR spectroscopy—a pilot study at 4 T. Radiology.

[36] Munzone E, Colleoni M (2015). Clinical overview of metronomic chemotherapy in breast cancer. Nat Rev Clin Oncol.

[37] NCCN. 2019. “Clinical practice guidelines: breast Cancer.” National Comprehensive Cancer Network 2019. https://www.nccn.org/professionals/physician_gls/pdf/breast_blocks.pdf. Accessed 18 May 2019.

[38] Newton K, Dixit VM (2012). Signaling in innate immunity and inflammation. Cold Spring Harb Perspect Biol.

[39] No K-s, Kwong R, Chou PH, Cerussi A (2008). Design and testing of a miniature broadband frequency domain photon migration instrument. J Biomed Opt.

[40] O’Sullivan TD, Cerussi AE, Cuccia DJ, Tromberg BJ (2012). Diffuse optical imaging using spatially and temporally modulated light. J Biomed Opt.

[41] Partridge SC, Zheng Z, Newitt DC, Gibbs JE, Chenevert TL, Rosen MA, Bolan PJ (2018). Diffusion-weighted MRI findings predict pathologic response in neoadjuvant treatment of breast cancer: the ACRIN 6698 multicenter trial. Radiology.

[42] Pasquier E, Kavallaris M, André N (2010). Metronomic chemotherapy: new rationale for new directions. Nat Rev Clin Oncol.

[43] Pham TH, Coquoz O, Fishkin JB, Anderson E, Tromberg BJ (2000). Broad bandwidth frequency domain instrument for quantitative tissue optical spectroscopy. Rev Sci Instrum.

[44] Rastogi P, Anderson SJ, Bear HD, Geyer CE, Kahlenberg MS, Robidoux A, Margolese RG (2008). Preoperative chemotherapy: updates of National Surgical Adjuvant Breast and Bowel Project Protocols B-18 and B-27. J Clin Oncol.

[45] Roblyer D, Ueda S, Cerussi A, Tanamai W, Durkin A, Mehta R, Hsiang D (2011). Optical imaging of breast cancer oxyhemoglobin flare correlates with neoadjuvant chemotherapy response one day after starting treatment. Proc Natl Acad Sci U S A.

[46] Sajjadi AY, Isakoff SJ, Deng B, Singh B, Wanyo CM, Fang Q, Specht MC (2017). Normalization of compression-induced hemodynamics in patients responding to neoadjuvant chemotherapy monitored by dynamic tomographic optical breast imaging (DTOBI). Biomed Optics Express.

[47] Tromberg BJ, Pogue BW, Paulsen KD, Yodh AG, Boas DA, Cerussi AE (2008). Assessing the future of diffuse optical imaging technologies for breast cancer management. Med Physics.

[48] Tromberg BJ, Zheng Z, Leproux A, O’Sullivan TD, Cerussi AE, Carpenter PM, Mehta RS (2016). Predicting responses to neoadjuvant chemotherapy in breast cancer: ACRIN 6691 trial of diffuse optical spectroscopic imaging. Cancer Res.

[49] Ueda S, Roblyer D, Cerussi A, Durkin A, Leproux A, Santoro Y, Xu S (2012). Baseline tumor oxygen saturation correlates with a pathologic complete response in breast cancer patients undergoing neoadjuvant chemotherapy. Cancer Res.

[50] Ueda S, Saeki T, Osaki A, Yamane T, Kuji I (2017). Bevacizumab induces acute hypoxia and cancer progression in patients with refractory breast cancer: multimodal functional imaging and multiplex cytokine analysis. Clin Cancer Res.

[51] Wanderley CW, Colon DF, Luiz JPM, Oliveira FF, Viacava PR, Leite CA, Pereira JA (2018). Paclitaxel reduces tumor growth by reprogramming tumor-associated macrophages to an M1- profile in a TLR4-dependent manner. Cancer Res.

[52] Yeh E, Slanetz P, Kopans DB, Rafferty E, Georgian-Smith D, Moy L, Halpern E, Moore R, Kuter I, Taghian A (2005). Prospective comparison of mammography, sonography, and MRI in patients undergoing neoadjuvant chemotherapy for palpable breast cancer. Am J Roentgenol.

[53] Zhu Q, Tannenbaum S, Kurtzman SH, DeFusco P, Ricci A, Vavadi H, Zhou F (2018). Identifying an early treatment window for predicting breast cancer response to neoadjuvant chemotherapy using Immunohistopathology and hemoglobin parameters. Breast Cancer Res.

