# Supplementary material for: Diffuse optical spectroscopic imaging reveals distinct early breast tumor hemodynamic responses to metronomic and maximum tolerated dose regimens
Source: Breast Cancer Res. 2020 Mar 13;22:29. doi: 10.1186/s13058-020-01262-1 (PMC7071774; doi:10.1186/s13058-020-01262-1)
Supplement: Supplementary file 1 — Post-hoc GEE Analysis. A) Post-hoc GEE Estimates of Oxyhemoglobin differences across Response of MTD patients on days 1-7. B) Post-hoc GEE Estimates of Oxyhemoglobin differences across Treatment of Responding patients on days 1-7. Significance is determined at Bonferroni corrected level of p<0.0036. [file 13058_2020_1262_MOESM1_ESM.pdf]

Responders Cohort: MTD vs MET

Days Postchemotherapy	Difference Estimate %HbO2	Standard Error	Z value	P Value
1	39.4548	11.9849	3.29	0.001
2	27.6914	15.5288	1.78	0.0745
3	45.1494	15.8659	2.85	0.044
4	29.1872	14.0117	2.08	0.0372
5	18.733	12.8497	1.46	0.1449
6	27.1901	14.8437	1.83	0.067
7	9.7234	15.4755	0.63	0.5298

Table S1A) Post-hoc GEE Estimates of Oxyhemoglobin differences across Treatment of Responding patients on days 1-7. Significance is determined at Bonferroni corrected level of $p<0.0036$.

MTD Cohort: Responders vs Non-Responders

Days Postchemotherapy	Difference Estimate %HbO2	Standard Error	Z value	P Value
1	48.7737	9.5129	5.13	<.0001
2	28.8053	14.3329	2.01	0.0059
3	48.897	15.0479	3.25	0.0012
4	38.9028	12.8756	3.02	0.0025
5	19.6682	16.6273	1.18	0.2369
6	38.1247	13.3738	2.85	0.044
7	25.3257	13.3915	1.89	0.0586

Table S1B) Post-hoc GEE Estimates of Oxyhemoglobin differences across Response of MTD patients on days 1-7. Significance is determined at Bonferroni corrected level of $p<0.0036$.
