# Supplementary figures and images for: Diffuse optical spectroscopic imaging reveals distinct early breast tumor hemodynamic responses to metronomic and maximum tolerated dose regimens
Source: Breast Cancer Res. 2020 Mar 13;22:29. doi: 10.1186/s13058-020-01262-1 (PMC7071774; doi:10.1186/s13058-020-01262-1)

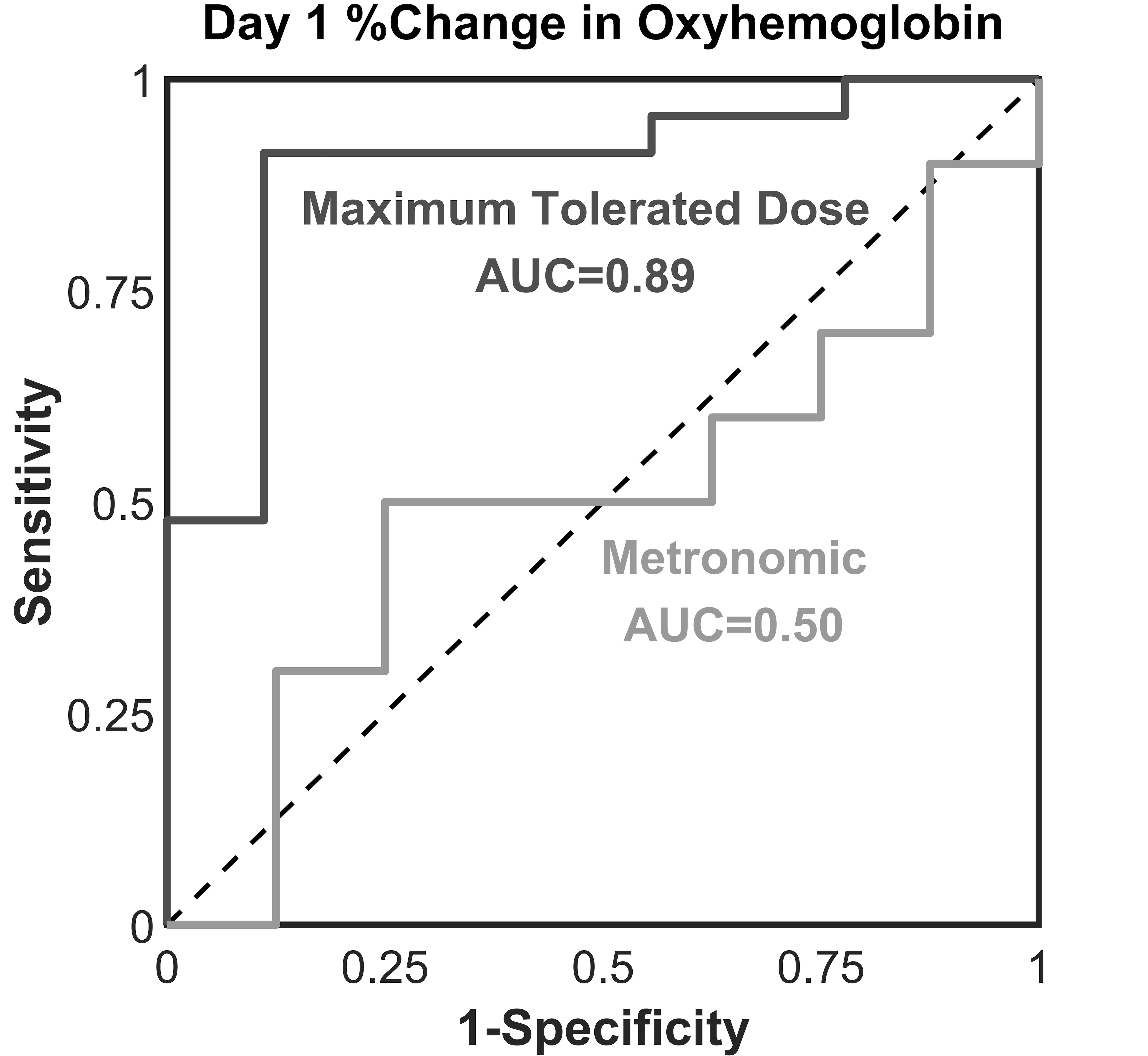

Supplement: Supplementary file 2 — Prognostic Accuracy of Oxyhemoglobin Flare. Receiver Operator Characteristic (ROC) Curve of percent change in oxyhemoglobin on day 1 postchemotherapy as a classifier for pathologic response (Responders vs Non-Responders) for both MTD (dark grey) and MET (light grey) with their corresponding area under the curve (AUC). [file 13058_2020_1262_MOESM2_ESM.png]

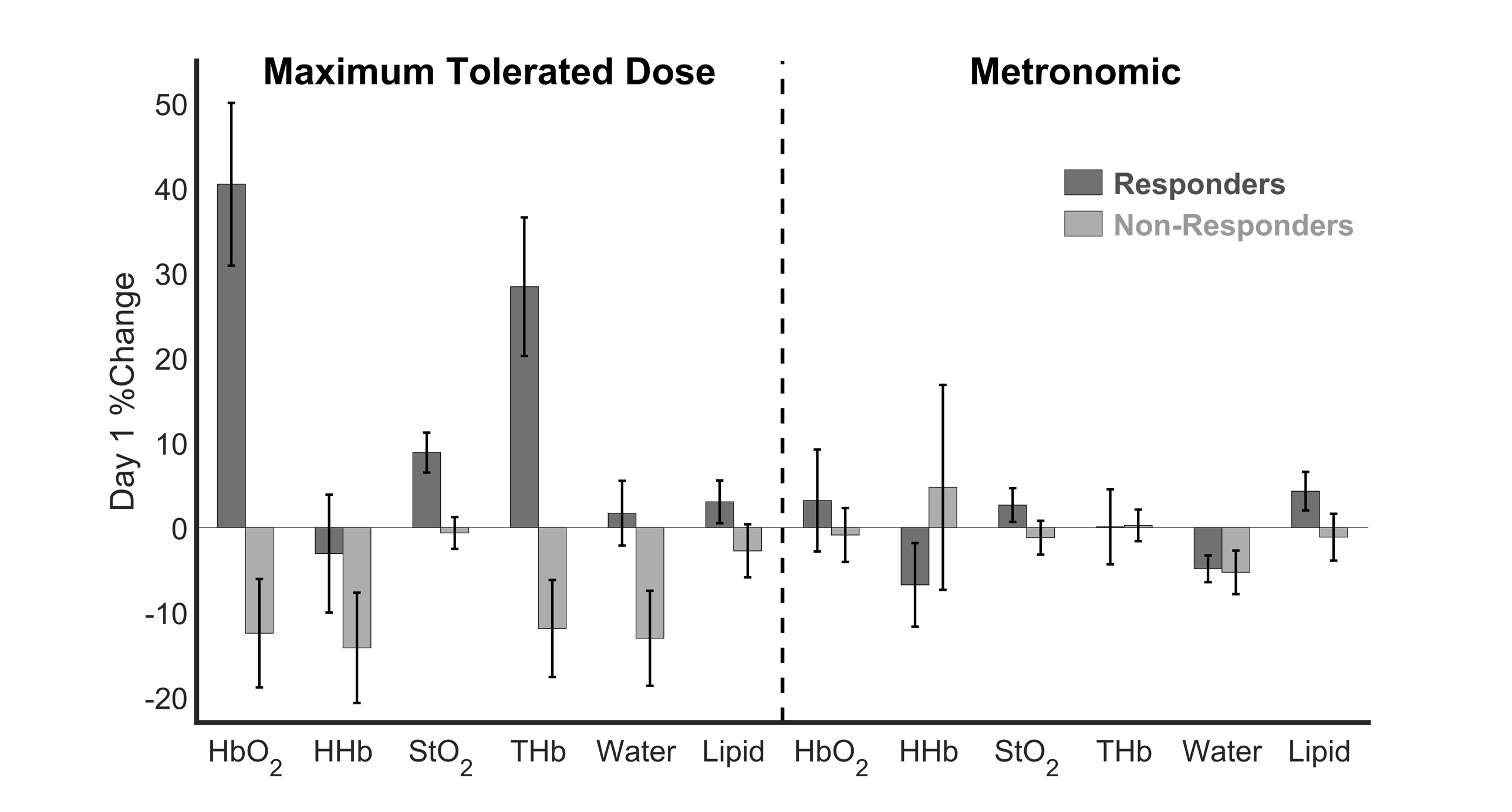

Supplement: Supplementary file 3 — Day 1 Hemodynamic Changes of DOSI-monitored tumors. Percent change during day 1 postchemotherapy in oxyhemoglobin, deoxyhemoglobin, oxygen saturation, total hemoglobin, water, and lipid separated by treatment: Maximum Tolerated Dose (left) and Metronomic (right) and pathologic response: Responders (Dark Grey) and Non-Responders (Light Grey). Error bars represent mean ± standard error. [file 13058_2020_1262_MOESM3_ESM.tif]
